# Facile Surface Modification of MgMn_2_O_4_ Positive-Electrode Material for Improving Cycle Performance of Magnesium Rechargeable Batteries

**DOI:** 10.1021/acsomega.2c06633

**Published:** 2022-12-09

**Authors:** Naoto Kitamura, Tomoya Imura, Naoya Ishida, Chiaki Ishibashi, Yasushi Idemoto

**Affiliations:** † Department of Pure and Applied Chemistry, Faculty of Science and Technology, Tokyo University of Science, 2641 Yamazaki, Noda, Chiba278-8510, Japan; ‡ Research Group for Advanced Energy Conversion, Research Institute for Science and Technology, Tokyo University of Science, 2641 Yamazaki, Noda, Chiba278-8510, Japan

## Abstract

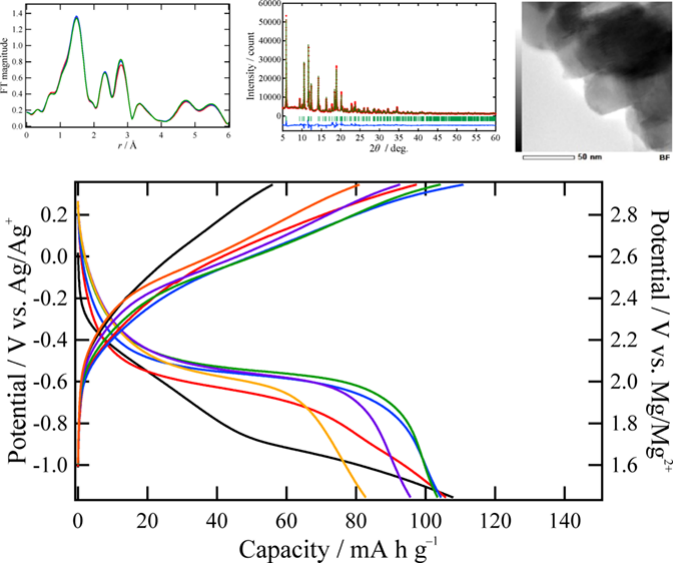

MgMn_2_O_4_ with a tetragonal spinel structure shows promise as a positive-electrode material in magnesium rechargeable batteries (MRBs), which have drawn considerable attention as post lithium-ion batteries. However, the material currently suffers from poor cycle performance. In this study, we attempt to improve the cycle performance of MgMn_2_O_4_ via the Zr modification of its particle surface. X-ray photoelectron spectroscopy and energy-dispersive X-ray spectroscopy demonstrate that the surface modification is successfully performed by immersing MgMn_2_O_4_ powder into a Zr-containing aqueous solution, followed by heat treatment. However, Zr segregation is observed at high Zr concentration. Furthermore, structural analyses using synchrotron X-rays indicate that the Zr modification has an influence on the bulk structure of the MgMn_2_O_4_ powder. The positive-electrode properties of the powders are investigated using discharge/charge cycle tests, which show that Zr modification can drastically improve the cycle performance and coulombic efficiency. These improvements are supposed to be due to suppression of an unexpected reaction by the Zr-surface modification and lower structural distortion after the modification. These findings clearly demonstrate the significant potential of surface modification as a method for obtaining high-performance MRBs.

## 1 Introduction

In recent decades, there has been an increasing emphasis on reducing environmental load and CO_2_ emissions by enhancing the usage efficiency of renewable energy. This has warranted the development of large rechargeable batteries that can store that energy. To accomplish this, many researchers have proposed various types of post lithium-ion batteries (LIBs) composed of abundant elements that are safer and have a higher energy density than the widely used LIBs. Magnesium rechargeable batteries (MRBs) are promising^1^ because Mg, generally used as a negative electrode, is more abundant and safer than Li. Moreover, MRBs can theoretically deliver much higher discharge capacities than LIBs because of a discharge/charge reaction involving insertion/deinsertion of divalent cations (Mg^2+^) accompanied by the redox of transition metal cations. This has prompted material research on MRB-positive electrodes, with several studies in the last decade successfully uncovering novel oxides that can function as positive electrodes. For example, Mg_1+*x*_*M*_2–*x*_O_4_ (*M* = transition metal) with a spinel structure,^2−22^ Mg*M*O_2_ with a rocksalt structure,^23^ ZnMnO_3_ with a deficient spinel structure,^24,25^ and magnesiated layered materials^26−28^ have been investigated, considering the fact that spinel and layered rocksalt structures provide ion diffusion paths in LIB electrode materials. Among these oxides, spinel-type MgMn_2_O_4_ with a high theoretical capacity (270 mA h g^–1^ for Mg insertion) is promising because it is composed only of abundant elements (Mg, Mn, and O) and can be synthesized by a simple inverse coprecipitation method with subsequent heat treatment.^2,3^ Indeed, MgMn_2_O_4_ can deliver high discharge capacities over 200 mA h g^–1^, but its cycle performance in anhydrous electrolytes is supposed to be insufficient for practical use.^4,5,8^ As is well known, in the case of LIBs and other rechargeable batteries, surface modification and partial substitution of the electrode powders are the good strategies to overcome this drawback.^29−36^ Currently, however, there are few reports on the surface modification of MRB electrode materials.^37−39^

In this work, we focused on MgMn_2_O_4_ as a positive-electrode material and performed surface modification on the powder to improve the cycle performance of the MRBs. ZrO_2_ modification of the positive-electrode materials in LIBs has been known to enhance electrochemical properties, such as the cycle performance;^29−33^ therefore, it was selected as the modification material for MgMn_2_O_4_ in this study. The surface properties of pristine and surface-modified MgMn_2_O_4_ were characterized by X-ray photoelectron spectroscopy (XPS) and energy-dispersive X-ray spectroscopy (EDX). The crystal structure (averaged structure) and local environment around Mn were also investigated using synchrotron X-rays to elucidate the effects of the Zr-modification process on the atomic configuration. Based on these results, the efficacy of surface modification of a spinel-type positive-electrode material using a Zr compound is demonstrated for the first time.

## 2 Experimental Section

To begin the MgMn_2_O_4_ synthesis, a precursor was prepared by a published inverse coprecipitation method.^2,3^ First, Mg(NO_3_)_2_ and Mn(NO_3_)_2_ were dissolved in pure water to prepare 1 mol dm^–3^ aqueous solutions of Mg and Mn. These solutions were mixed in an appropriate proportion, and then the mixed solution was added to a Na_2_CO_3_ aqueous solution at 70 °C with stirring. The precipitate was washed with hot water and dried at 100 °C. MgMn_2_O_4_ was obtained by firing the dried precursor in air at 650 °C for 24 h. To modify the surface of the MgMn_2_O_4_ powder, 3.0 mg of zirconium(IV) acetate hydroxide was dissolved in 30 cm^3^ of pure water, and then 300 mg of MgMn_2_O_4_ was added to the solution.^29,30^ The zirconium(IV) acetate hydroxide to MgMn_2_O_4_ weight ratio was 1 wt %. After stirring for 24 h, the obtained powder after filtration was heat-treated in air at 600 °C for 5 h. The surface-modified powder is hereafter referred to as MMO-Zr1. Aqueous Zr solutions with 6.0 and 9.0 mg of zirconium(IV) acetate hydroxide were also prepared and used to modify the surface of MgMn_2_O_4_ in the same manner. These powders were denoted as MMO-Zr2 and MMO-Zr3, respectively.

The phases of the pristine MgMn_2_O_4_ and surface-modified samples were preliminarily identified by a laboratory X-ray diffractometer with Cu *K*α radiation (Empyrean, PANalytical), and the metal compositions were estimated by inductively coupled plasma atomic emission spectroscopy (ICP-AES; ICPE-9000, Shimadzu). The particle morphologies and elemental distributions in the samples were investigated using scanning transmission electron microscopy (STEM) and EDX (JEM-2100F, JET-2300 T, JEOL). Surface atomic compositions and electronic states were studied using XPS (AXIS-NOVA, Kratos Analytical) with Al *K*α radiation. The averaged structures of the samples were refined by the Rietveld method using synchrotron X-ray diffraction (XRD) patterns (λ = 0.5 Å) measured at BL19B2 (SPring-8). Structural refinements were performed using the Rietan-FP software.^40^ Electronic and local structures were also elucidated by analyzing the X-ray absorption fine structure (XAFS) spectra at the Mn *K*-edge recorded at BL14B2 (SPring-8) using a transmission method. The X-ray absorption near-edge structure (XANES) and extended XAFS (EXAFS) obtained in the experiments were analyzed using the Athena program.^41^

To investigate the positive-electrode properties of the pristine and surface-modified MgMn_2_O_4_, galvanostatic discharge/charge cycle tests were performed at 90 °C using a three-electrode cell. To prepare the positive electrode, a mixture of each sample, Super C65, and PTFE in a weight ratio of 5:5:1 was pressed onto an Al mesh. The electrolyte was 1.0 mol dm^–3^ Mg[TFSI]_2_ in triglyme. The negative electrode was Mg alloy (Mg: Al: Zn = 96:3:1), denoted as AZ31, because AZ31 has better ductility and shows more uniform Mg dissolution/deposition than pure Mg.^42^ Due to the negative-electrode problem, Ag wire was used as a reference electrode, and the potential vs Ag/Ag^+^ was converted to that vs Mg/Mg^2+^ according to the literature.^43^ The current density was 5 mA g^–1^, and the cut-off potentials were −1.155 and 0.345 V vs Ag/Ag^+^. The capacity cut-off (time for discharging and charging) was also set at 300 mA h g^–1^ to eliminate contributions from unexpected reactions. The assembled cells were initially discharged, considering the following positive-electrode reaction^9,16,37−39^
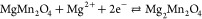
1where Mg_2_Mn_2_O_4_ has a rocksalt structure because the discharge/charge reaction can be considered to occur by a push-out process with the spinel-to-rocksalt transition.^4^

## 3 Results and Discussion

### 3.1 Metal Compositions and Surface Properties

**Figure 1:**
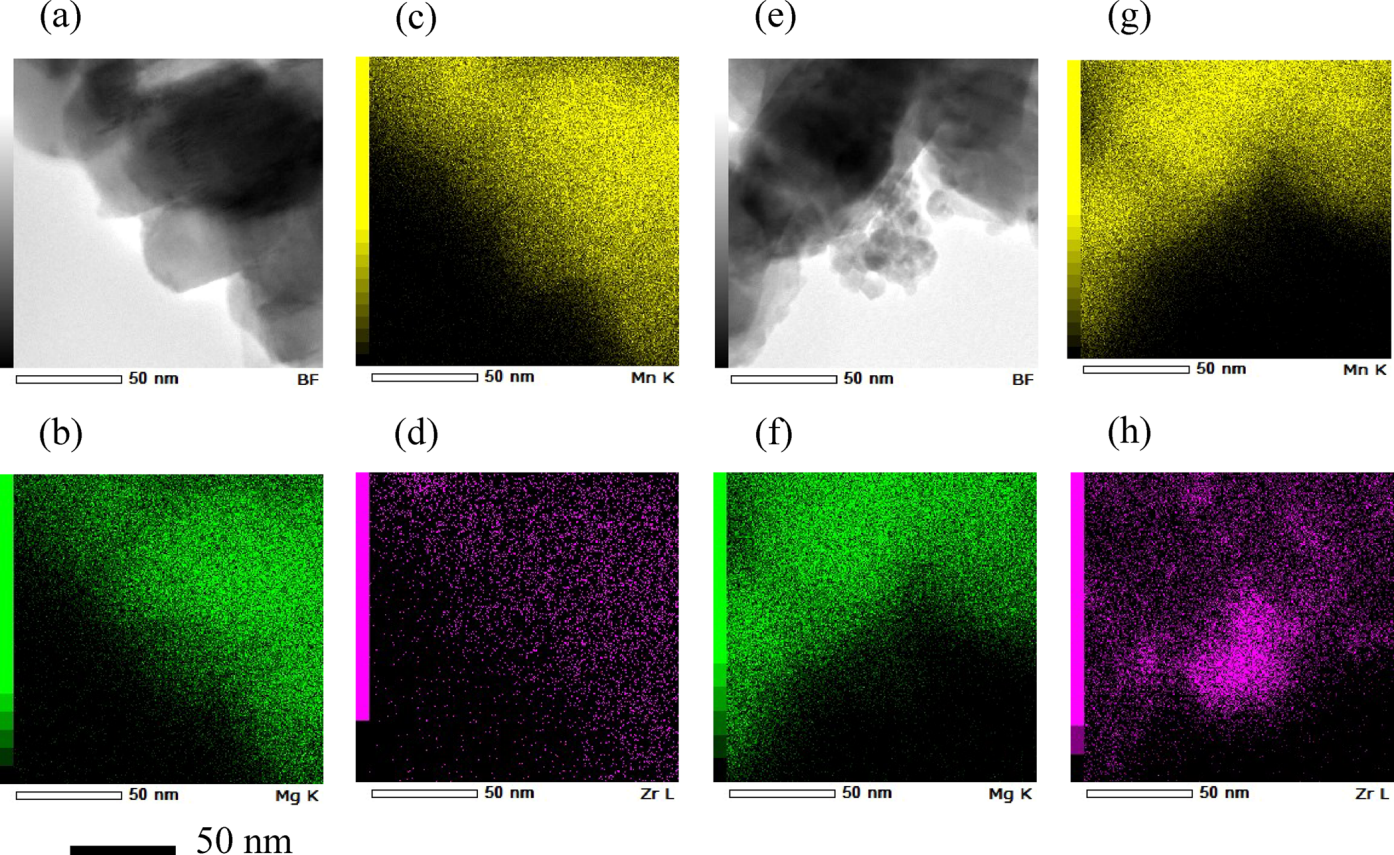
STEM-EDX images of (a–d) MMO-Zr1 and (e–h) MMO-Zr3: (a,e) Bright field (BF)-STEM image, (b,f) Mg mapping, (c,g) Mn mapping, and (d,h) Zr mapping.

The XRD (Figure S1) and ICP-AES results confirm that the MgMn_2_O_4_ synthesized in this study has a tetragonal spinel structure (space group: *I*4_1_/*amd*) with an analytical metal-composition ratio of Mg:Mn = 0.957(5):2.043(5); this is almost equal to the nominal ratio (1:2). Further, the averaged structure is maintained even after surface modification with the Zr compound (Figure S1). The averaged structures are discussed in detail in the next subsection. To elucidate the surface states after modification, STEM-EDX analysis was performed, and the results are presented in Figure 1. As can be seen in this figure, Zr is homogeneously distributed in MMO-Zr1, whereas it segregates to the surface of the particles in MMO-Zr3. For a quantitative evaluation, Table 1 lists the metal compositions of the surfaces of the specimens, with the selected areas presented in Figure S2. We also investigated Na contents by STEM-EDX because Na_2_CO_3_ was used in the synthetic process, but the contents are negligible [Figure S2(c)–(f)]. By comparing the compositions of the specific areas and whole images (Table 1), in the case of MMO-Zr3, it is clear that the Zr compositions of the areas 2–4 are much higher than that of the whole image and then higher than the compositions of main components (Mg and Mn). This result demonstrates that Zr segregation to the surface becomes significant with increasing Zr content. In other words, the surface modification with the Zr compound is homogeneously performed only when the Zr concentration in the treatment solution is less than 3 wt %.

**Table 1:** Atomic Compositions of MMO-Zr1 and MMO-Zr3 Studied by STEM-EDX^a^

sample	area	Mg	Mn	Zr
MMO-Zr1	area 1	0.89	2	0.01
	whole	1.04	2	0.005
MMO-Zr3	area 2	1.13	2	5.33
	area 3	1.08	2	2.03
	area 4	1.04	2	12.32
	whole	1.02	2	0.45

a The compositions were normalized under the assumption that the Mn composition was equal to 2 in both samples. The selected areas are presented in Figure S2, and the whole values are estimated from the whole images.

**Figure 2:**
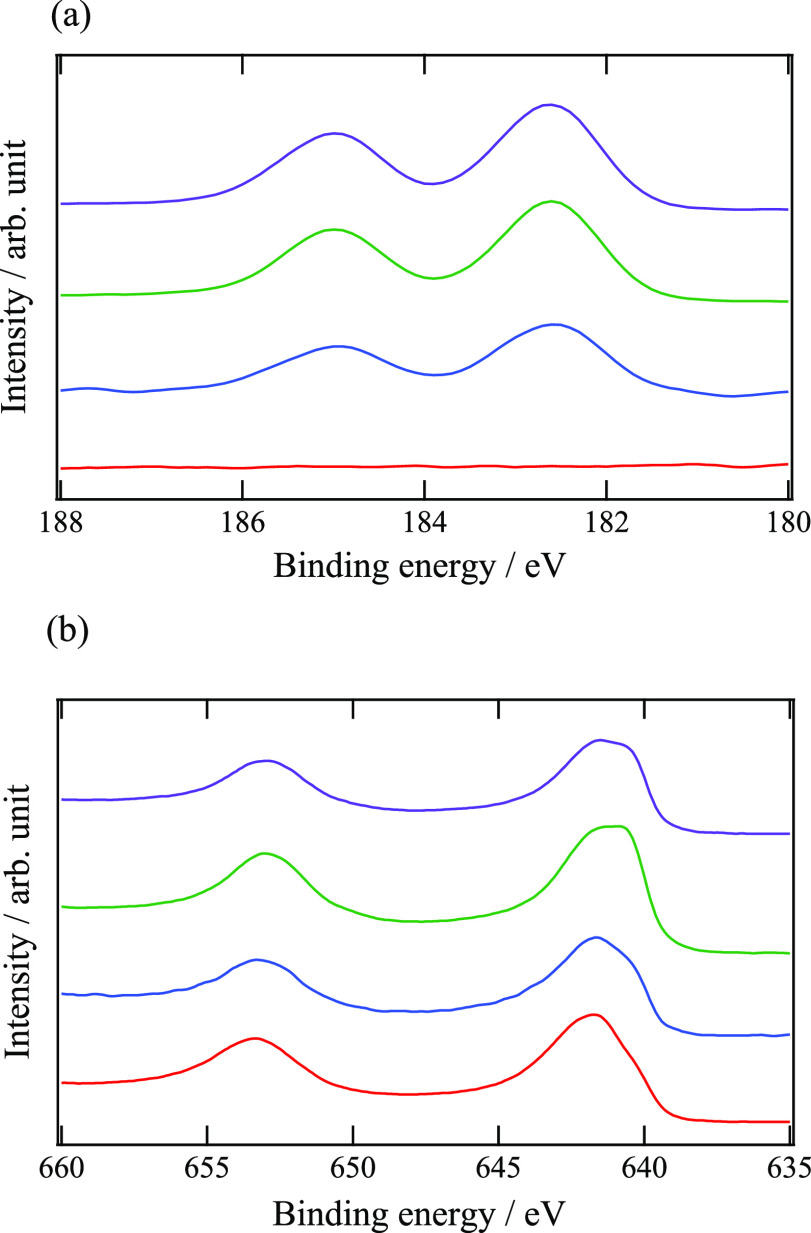
XPS profiles of the pristine and Zr-modified MgMn_2_O_4_: (a) Zr 3d and (b) Mn 2p. The red, blue, green, and purple lines represent the pristine MgMn_2_O_4_, MMO-Zr1, MMO-Zr2, and MMO-Zr3, respectively.

The surface properties were also investigated by XPS analysis, and the spectra of Zr 3d and Mn 2p are presented in Figure 2a,b, respectively. After surface modification, Zr can be detected, regardless of the Zr concentration in the treatment. This result is consistent with that of the STEM-EDX analysis mentioned above. It is also shown that peaks at Mn 2p shift toward a lower binding energy after Zr modification (Figures 2b and S3). This change in the Mn electronic state implies that Mn on the surface is slightly reduced by the modification. In other words, the modification process affects the host structure of MgMn_2_O_4_ in addition to the surface of the MgMn_2_O_4_ powder. This effect on the bulk structure is likely caused by the heat treatment at 600 °C after immersion of the powder in the Zr-containing aqueous solution because a similar effect was previously reported for LIB positive-electrode materials.^31,32^

### 3.2 Bulk Properties: Electronic, Averaged, and Local Structures

**Figure 3:**
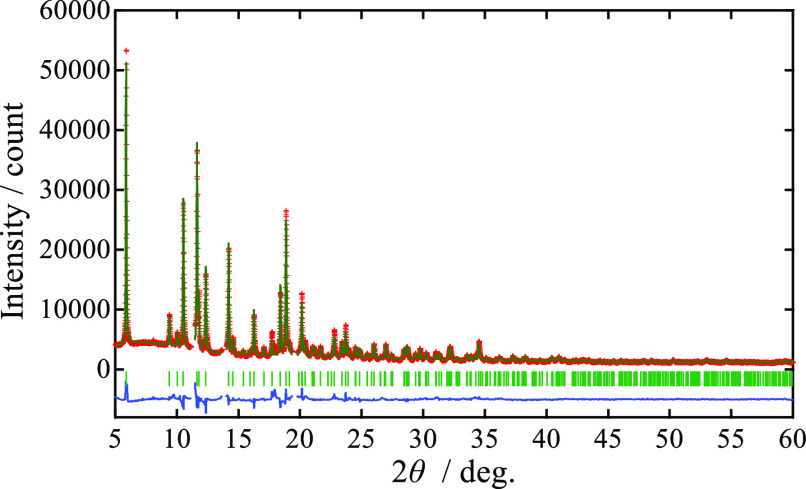
Rietveld refinement pattern of MMO-Zr1. The red crosses show observed intensities, and the green solid line represents calculated intensities. The vertical bars indicate positions of allowed Bragg reflections. The blue curve at the bottom is the difference between the observed and calculated intensities at the same scale.

For a detailed investigation of the bulk structures, synchrotron XRD patterns were obtained and Rietveld refinements were performed. The Rietveld refinement patterns and refined structural parameters of MMO-Zr1 are shown in Figure 3 and Table 2, respectively, and the parameters of the other samples are listed in Table S2. Since the main phase can be attributed to the tetragonal spinel phase (Figure S1), the space group was assumed to be *I*4_1_/*amd*, according to the literature.^5^ In the refinement, Zr was not considered because of the very low Zr content (Table 1). Since a previous work suggested a cation mixing (inversion) of Mg and Mn especially at elevated temperature,^21^ Mg and Mn occupancies at the tetrahedral (4a) and octahedral (8d) sites were refined while maintaining the metal ratio as an analytical value by ICP-AES. As a result, the Mg occupancy at the octahedral site became slightly negative in the refinement process, indicating that the octahedral site is not occupied by Mg. Therefore, we fixed this value as zero to avoid meaningless analyses. As can be seen in Figure 3, the averaged structure could be refined under these hypotheses, and thus it is demonstrated that Mg and Mn exist basically at the tetrahedral and octahedral sites, respectively, as reported previously.^20,44^ Distortions of the tetrahedra (*M*O_4_) and octahedra (*M*O_6_) were calculated from the refined structural parameters. Table 3 summarizes the polyhedral volumes, quadratic elongations (λ), and bond-angle variances (σ^2^), which are indices of the polyhedral distortions.^45^ In all the samples, the distortion of *M*O_6_ is significant because the octahedral site is occupied by Mn^3+^ with the Jahn–Teller effect. It is noteworthy that the distortion indices, especially those of the tetrahedra, are smaller in the surface-modified samples than in the pristine state. It is also shown that the volume of the tetrahedron decreases with modification. These results demonstrate that the modification process has a slight influence on the bulk structure of MgMn_2_O_4_. Considering these changes in the tetrahedral site, it is inferred that some of the Zr is incorporated into the bulk structure, with a small amount of transition metal cations migrating to the tetrahedral site during the surface modification process.

**Table 2:** Refined Structural Parameters of MMO-Zr1 with the Tetragonal Spinel Structure (S.G.: *I*4_1_/*amd*)^a^

atom	site	*x*	*y*	*z*	*B*/Å^2^	*g*
Mg	4a	0	1/4	7/8	0.52(4)	0.957
Mn1	4a	= Mg(*x*)	= Mg(*y*)	= Mg(*z*)	= Mg(*B*)	0.043
Mn2	8d	0	1/2	1/2	0.46(2)	1
O	16h	0	0.4724(3)	0.2573(2)	0.34(3)	1

a *B* and *g* represent the atomic displacement parameter and site occupancy, respectively. *R* factors are *R*_wp_ = 6.59%, *R*_p_ = 4.77%, and *R*_e_ = 1.98%. Lattice parameters are *a* = 5.7206(1) Å and *c* = 9.3007(3) Å.

**Table 3:** Polyhedral Volumes, Quadratic Elongations (λ), and Bond-Angle Variances (σ^2^) of (a) *M*O_4_ and (b) *M*O_6_

sample	volume/Å^3^	λ	σ^2^/deg.^2^
(a)			
pristine	4.131	1.004	17.28
MMO-Zr1	4.140	1.004	15.36
MMO-Zr2	4.098	1.004	15.64
MMO-Zr3	4.025	1.004	14.67
(b)			
pristine	10.92	1.022	34.52
MMO-Zr1	10.91	1.022	35.06
MMO-Zr2	10.98	1.022	32.26
MMO-Zr3	11.11	1.021	27.34

**Figure 4:**
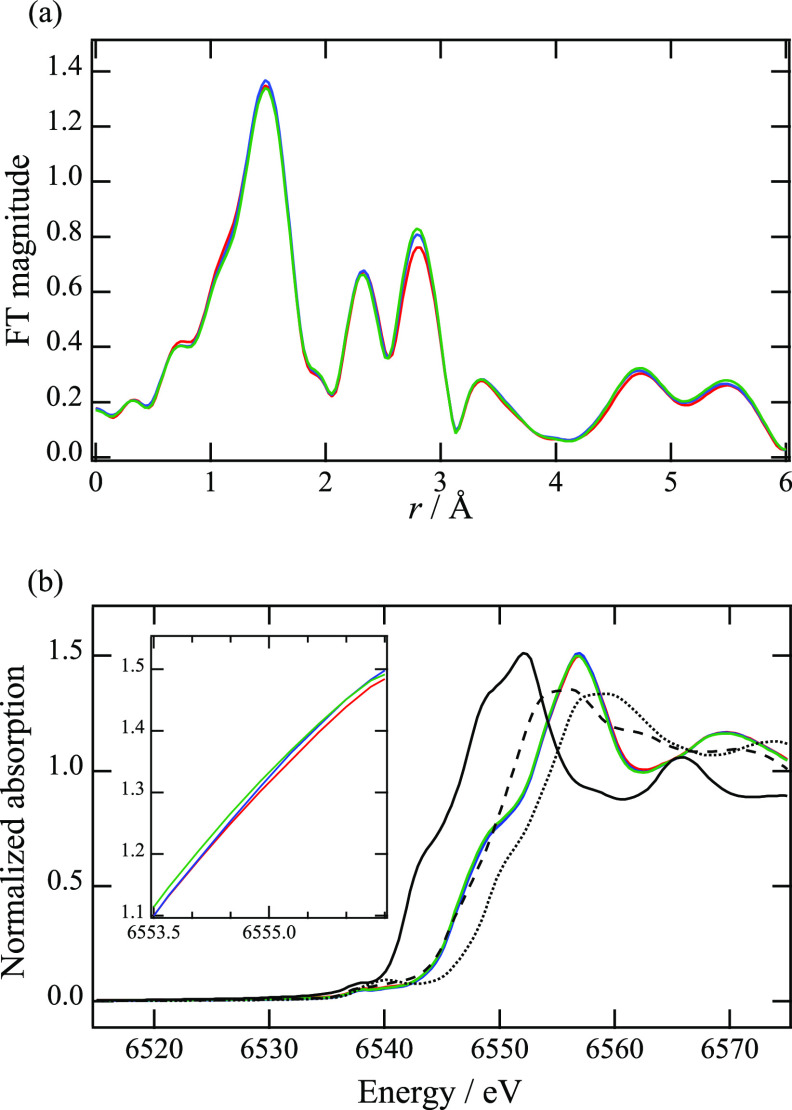
(a) FT magnitudes of EXAFS oscillations and (b) XANES spectra at Mn *K*-edge with some reference materials. The red, blue, and green lines represent the pristine MgMn_2_O_4_, MMO-Zr1, and MMO-Zr2, respectively. The solid, broken, and dotted lines represent MnO, Mn_2_O_3_, and MnO_2_, respectively.

To elucidate the change in the atomic configuration from the perspective of the local environment around Mn, Fourier transforms (FT) of the EXAFS oscillations in the *k* range from 3 to 12 Å^–1^ using *k*^2^ weighting were obtained, with the results presented in Figure 4a. The FT magnitudes indicate that the peak intensity at the third coordination shell, corresponding to the correlation between Mn and the metals at the tetrahedral site, increases after modification. This change is likely related to the relaxation of the structural distortion and/or metal migration suggested by the Rietveld refinement (Table 3).

The change in the electronic state of Mn was also investigated using XANES, and the spectra are shown in Figure 4b. In contrast to the XPS spectra (Figure 2b), the electronic structural change due to surface modification is essentially negligible, but the absorption edge slightly shifted toward a lower energy. This result indicates that the influence of the modification on the reduction of Mn^3+^ is less significant in the bulk (Figure 4b) than on the particle surface (Figure 2b), suggesting that the effect of Zr modification follows a gradient in the MgMn_2_O_4_ particles. According to a previous study,^31^ this gradual change is likely induced by the heat treatment at a relatively high temperature (600 °C) in the surface modification process.

### 3.3 Positive-Electrode Properties

**Figure 5:**
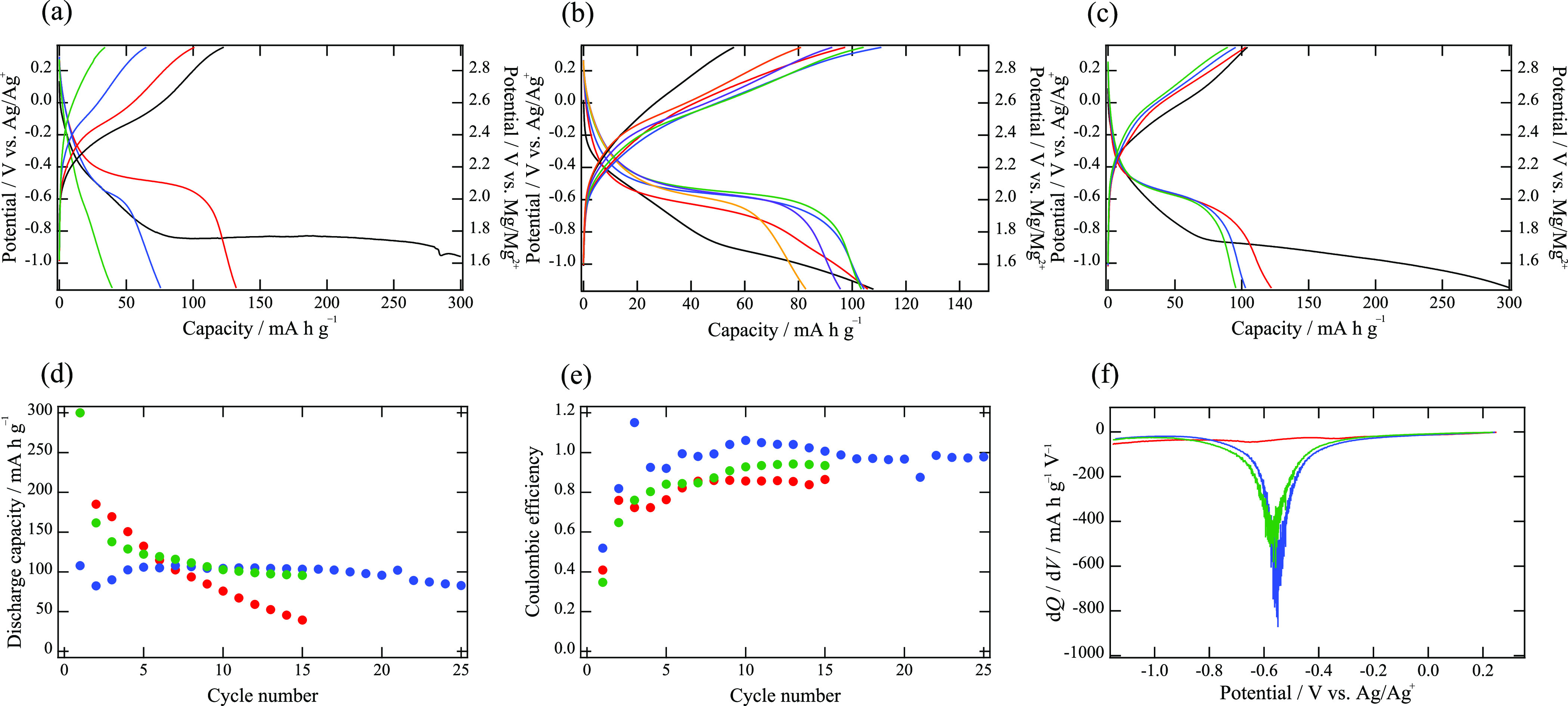
Discharge and charge curves of (a) pristine MgMn_2_O_4_, (b) MMO-Zr1, and (c) MMO-Zr2. The black, red, blue, green, purple, and orange lines represent the curves at 1st, 5th, 10th, 15th, 20th, and 25th cycles, respectively. (d) Discharge capacities and (e) coulombic efficiencies (charge capacity/discharge capacity) as a function of the cycle number. The red, blue, and green circles represent the pristine MgMn_2_O_4_, MMO-Zr1, and MMO-Zr2, respectively. (f) Derivative plots of d*Q*/d*V* at the 15th cycle. The color scheme is the same as in Figure 5d,e.

Figure 5a–c shows the discharge and charge curves of pristine MgMn_2_O_4_, Zr-modified MMO-Zr1, and MMO-Zr2, respectively. According to previous theoretical studies,^44,46^ Mg deinsertion and insertion with redox of Mn^3+^/Mn^4+^ can occur around 2.8 V vs Mg/Mg^2+^ in the case of MgMn_2_O_4_. As for the acid-treated MgMn_2_O_4_ (demagnesiated Mg_*x*_Mn_2_O_4_),^20^ it was also suggested that a pseudoplateau due to the oxidation up to Mn^4+^ was observed at ca. 2.5 V vs Mg/Mg^2+^. Such a contribution of Mn^3+^/Mn^4+^ redox was also confirmed in a cubic Mg_*x*_Mn_2_O_4_ with vacancy at the tetrahedral site, in which Mn was partially oxidized from trivalent to tetravalent.^8^ In contrast with these works, no pseudoplateau was observed at the voltage region of the discharge/charge curves in this work. This may be because Mn is trivalent in all the synthesized samples (Figure 4b). Therefore, it can be considered that the discharge capacities of the pristine MgMn_2_O_4_, Zr-modified MMO-Zr1, and MMO-Zr2 (Figure 5a–c) is due to Mg insertion with Mn^2+^/Mn^3+^ redox as eq 1. Indeed, the pseudoplateau around 2 V vs Mg/Mg^2+^ observed here is well consistent with previous studies which reported Mg insertion into MgMn_2_O_4_ with Mn^2+^/Mn^3+^ redox.^9,16,37−39^

In the pristine sample (Figure 5a), the initial discharge capacity is large, but the capacity considerably deteriorates as the number of cycles increases. In contrast, MMO-Zr1 maintains its discharge capacity even after 20 cycles, although its initial capacity is lower than that of the pristine sample. This result demonstrates that surface modification with the Zr compound can drastically improve the cycle performance of MgMn_2_O_4_. MMO-Zr2 also exhibits better cycle performance than the pristine sample, but its properties seem to be slightly inferior to those of MMO-Zr1. This might be due to an excess of Zr in MMO-Zr2, as Zr segregation is observed in MMO-Zr3 (Figure 1).

Figure 5d,e shows the discharge capacities and coulombic efficiencies (ratio of the charge capacity to the discharge capacity) as a function of the cycle number. As described above, MMO-Zr1 exhibits a good cycle performance and its coulombic efficiency approaches unity. This means that reactions at the positive electrode, excluding eq 1, could be suppressed by Zr modification. It is also likely that the lower crystal distortion in the Zr-modified sample makes the crystal structure stable and then improves the cycle performance. Therefore, it can be concluded that the Zr coating and/or the bulk structure change, especially near the surface, plays an important role in the improvement of the cycle performance.

To compare electrode properties after the cycles, the differential functions of the discharge capacities at the 15th cycle with the potential, d*Q*/d*V* (*Q*: capacity), are presented in Figure 5f. The surface modification of MgMn_2_O_4_ causes the peak intensity to become more prominent and the peak position to shift toward a slightly higher potential. These differences indicate higher capacities and lower overpotentials for the modified samples. Considering these results, post-treatments of electrode materials, such as the surface modification proposed here, can be regarded as a promising way to improve the cycle performance of MRBs.

## 4 Conclusions

In this work, the surface of the MgMn_2_O_4_ powder with a tetragonal spinel structure was modified using a Zr aqueous solution, and the surface and bulk properties of the modified powder were investigated. The positive-electrode properties of the samples were studied using discharge/charge cycle tests. It was noted that surface modification by the Zr compound without the occurrence of Zr segregation was only successful when the Zr concentration was low. Based on the average and local structural analyses, it was suggested that not only the surface state but also the bulk structure of MgMn_2_O_4_ varies after surface modification. Further, the discharge/charge cycle tests revealed that the cycle performance and coulombic efficiency can be significantly improved by Zr modification. These improvements are considered as the effects of the Zr modification, that is, suppression of an unexpected reaction by the Zr-surface modification and lower structural distortion after the modification. These findings shed new light on MgMn_2_O_4_-based positive-electrode materials for MRBs.

## Supporting Information Available

X-ray diffraction patterns, STEM images, EDX spectra, XPS spectra, and structural parameters refined by the Rietveld method of the pristine and surface-modified MgMn_2_O_4_ (PDF)
