# Supplementary material for: Facile Surface Modification of MgMn2O4 Positive-Electrode Material for Improving Cycle Performance of Magnesium Rechargeable Batteries
Source: ACS Omega. 2022 Dec 9;7(50):46915–21. doi: 10.1021/acsomega.2c06633 (PMC9774335; doi:10.1021/acsomega.2c06633)
Supplement: Supplementary file 1 — ao2c06633_si_001.pdf [file ao2c06633_si_001.pdf]

Supporting information

Facile Surface Modification of $MgMn_2O_4$ Positive-Electrode Material for Improving Cycle Performance of Magnesium Rechargeable Batteries

Naoto Kitamura,[*a, b] Tomoya Imura,[a] Naoya Ishida,[a] Chiaki Ishibashi,[a] Yasushi Idemoto[a, b]

a. Department of Pure and Applied Chemistry, Faculty of Science and Technology, Tokyo University of Science, 2641 Yamazaki, Noda, Chiba 278-8510, Japan.

b. Research Group for Advanced Energy Conversion, Research Institute for Science and Technology, Tokyo University of Science, 2641 Yamazaki, Noda, Chiba 278-8510, Japan

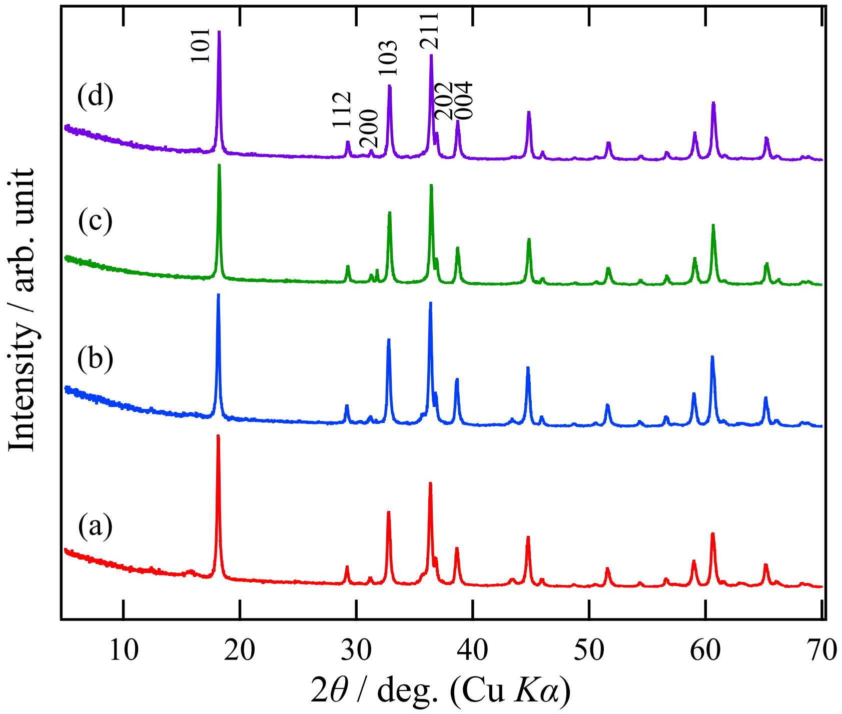

Figure S1. X-ray diffraction (XRD) patterns of (a) pristine $MgMn_2O_4$, (b) MMO-Zr1, (c) MMO-Zr2, and (d) MMO-Zr3.

Table S1. Crystallite sizes of the samples calculated from the XRD patterns (Figure S1) with a Sheller equation.

Sample	Crystallite size / nm
MMO	31
MMO-Zr1	32
MMO-Zr2	34
MMO-Zr3	33

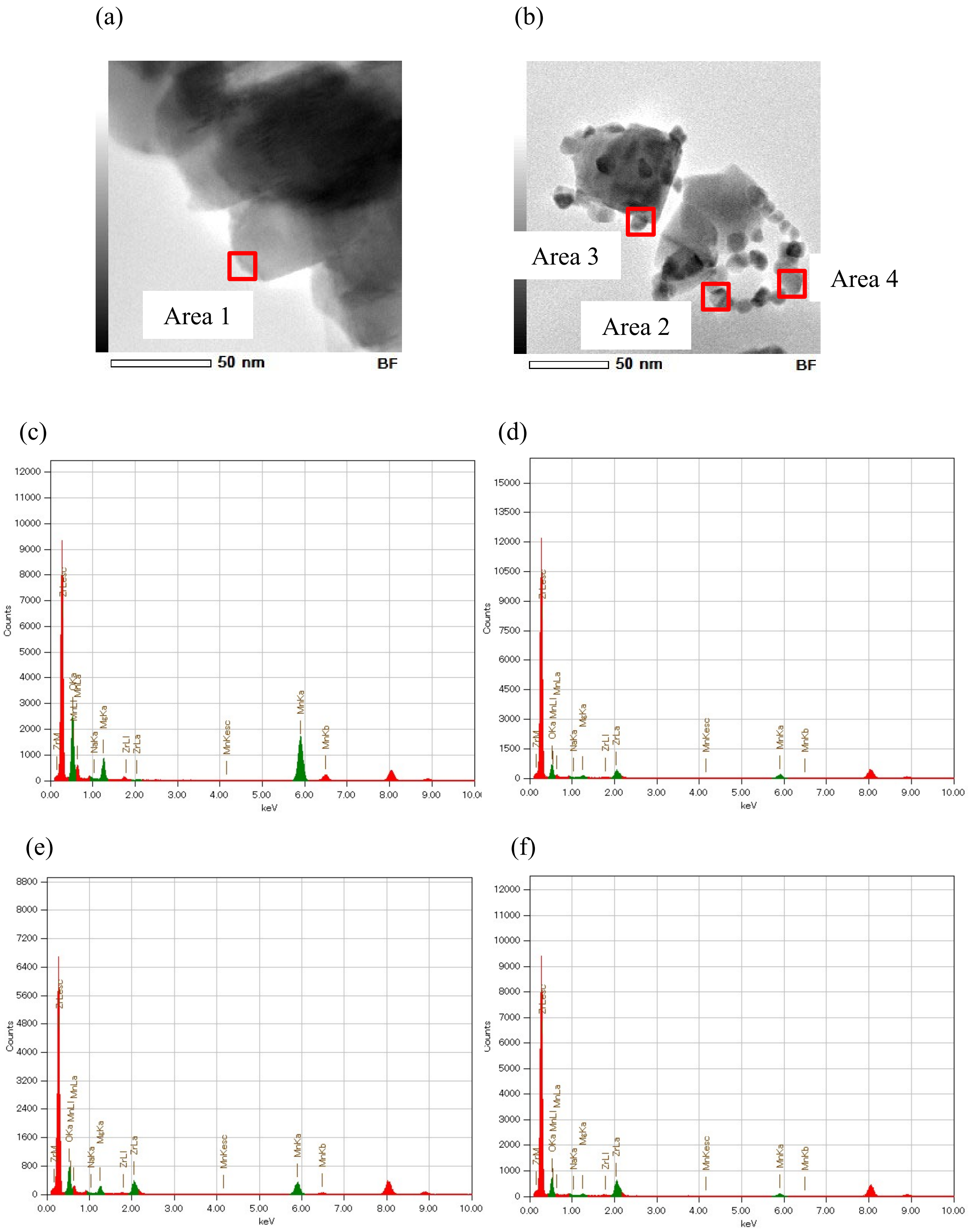

Figure S2. STEM images of (a) MMO-Zr1 an d (b) MMO-Zr3. EDX spectra of (c) Area 1, (d) Area 2, (e) Area 3, and (f) Area 4.

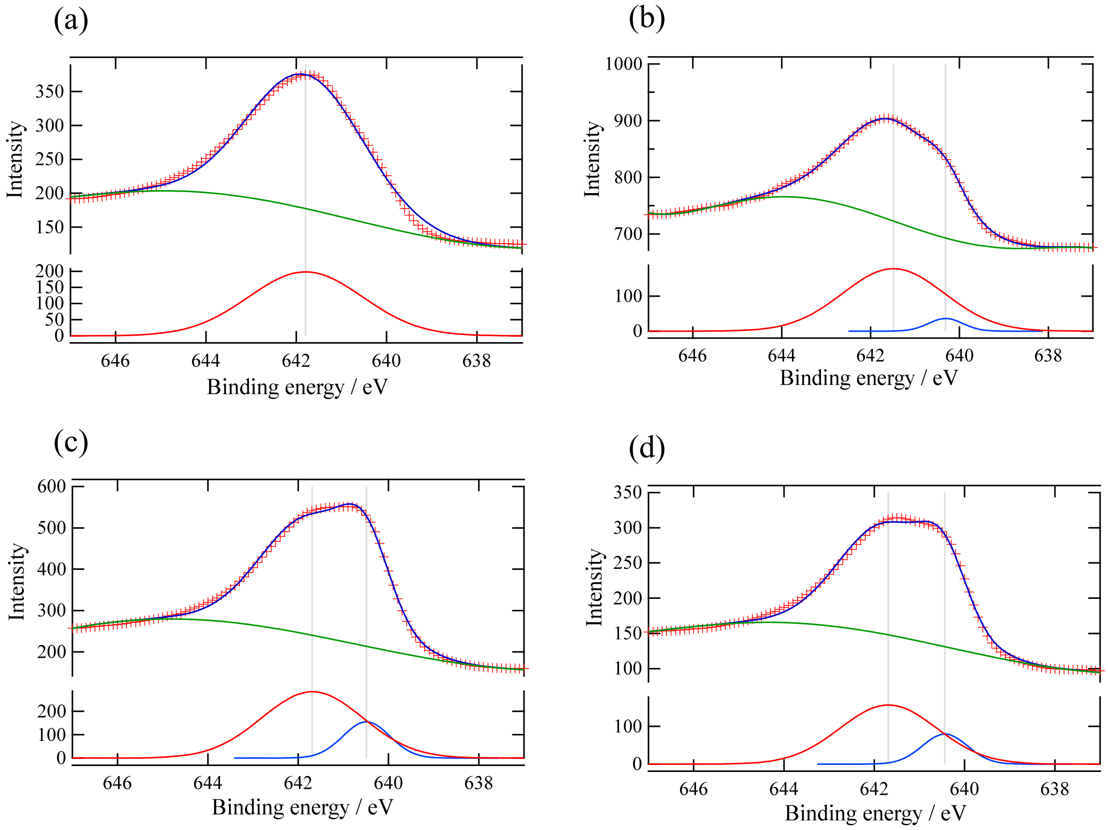

Figure S3. Fitting results of XPS profiles (Mn 2*p*) of the pristine and Zr-modified $MgMn_2O_4$. (a) pristine $MgMn_2O_4$, (b) MMO-Zr1, (c) MMO-Zr2, and (d) MMO-Zr3. The plus symbols and blue lines represent the experimental data and the overall fitting, respectively. The green lines represent the fitting of the background. The bottom patterns represent the deconvoluted peaks: Mn^{3+}, red; Mn^{2+}, blue.

Table S2. Refined structural parameters of (a) the pristine $MgMn_2O_4$, (b) MMO-Zr2, and (c) MMO-Zr3 with the tetragonal spinel structure (S. G.: $I4_1/amd$). B and g represent the atomic displacement parameter and site occupancy, respectively.

(a) R factors are R_{wp} = 8.38 %, R_p = 5.87 %, and R_e = 2.18 %. Lattice parameters are a = 5.7201(1) Å and c = 9.3007(3) Å.

Atom	Site	*x*	*y*	*z*	B / Å^2	*g*
Mg	4*a*	0	1/4	7/8	0.64(4)	0.957
Mn1	4*a*	= Mg(*x*)	= Mg(*y*)	= Mg(*z*)	= Mg(*B*)	0.043
Mn2	8*d*	0	1/2	1/2	0.50(2)	1
O	16*h*	0	0.4730(3)	0.2577(2)	0.42(3)	1

(b) R factors are R_{wp} = 7.78 %, R_p = 5.54 %, and R_e = 2.11 %. Lattice parameters are a = 5.7196(1) Å and c = 9.3008(2) Å.

Atom	Site	*x*	*y*	*z*	B / Å^2	*g*
Mg	4*a*	0	1/4	7/8	0.43(3)	0.957
Mn1	4*a*	= Mg(*x*)	= Mg(*y*)	= Mg(*z*)	= Mg(*B*)	0.043
Mn2	8*d*	0	1/2	1/2	0.42(2)	1
O	16*h*	0	0.4733(3)	0.2570(2)	0.37(3)	1

(c) R factors are R_{wp} = 8.62 %, R_p = 5.51 %, and R_e = 2.12 %. Lattice parameters are a = 5.7202(1) Å and c = 9.3033(2) Å.

Atom	Site	*x*	*y*	*z*	B / Å^2	*g*
Mg	4*a*	0	1/4	7/8	0.12(3)	0.957
Mn1	4*a*	= Mg(*x*)	= Mg(*y*)	= Mg(*z*)	= Mg(*B*)	0.043
Mn2	8*d*	0	1/2	1/2	0.36(2)	1
O	16*h*	0	0.4748(3)	0.2559(2)	0.28(3)	1
